# Effects of a synergic interaction between magnesium sulphate and ketamine on the perioperative nociception in dogs undergoing tibial plateau leveling osteotomy: a pilot study

**DOI:** 10.3389/fvets.2024.1453673

**Published:** 2024-10-30

**Authors:** Margherita Galosi, Luca Pennasilico, Angela Palumbo Piccionello, Federica Serino, Francesca Tosi, Sara Sassaroli, Valentina Riccio, Alessio Angorini, Alberto Salvaggio, Caterina Di Bella

**Affiliations:** School of Bioscience and Veterinary Medicine, University of Camerino, Camerino, Italy

**Keywords:** magnesium sulphate, ketamine, analgesia, orthopedic surgery, dogs

## Abstract

**Introduction:**

Magnesium Sulphate (MgSO_4_) is commonly used in human medicine for the management of perioperative pain in different types of procedures. However, in veterinary medicine, the use of MgSO_4_ has not been evaluated for its analgesic efficacy in dogs, which has generated conflicts of opinion in this area of veterinary anesthesiology. The aim of this study was to evaluate the perioperative analgesic efficacy of MgSO_4_ in combination with Ketamine in dogs undergoing Tibial Plateau Leveling Osteotomy (TPLO). Our hypothesis is that MgSO_4_ plus ketamine have a synergistic action in the management of intra-and postoperative pain.

**Methods:**

Twenty adult mixed breed dogs with average age 5.9 ± 2.6 years and weight 27.8 ± 9.2 kg were included in this prospective, clinical, randomized study. Dogs were randomly assigned to two groups. The MK group received ketamine (0.5 mg/kg as starting bolus followed by continuous infusion rate at 1 mg/kg/h). At the end of the ketamine bolus, MgSO_4_ (50 mg/kg over 15 min) was administered by the same route, followed by a constant rate infusion (CRI) at 15 mg/kg/h, IV. K group received a bolus of ketamine followed by a CRI at the same dosage described in MK group. Main cardiorespiratory parameters were recorded 10 min before the start of surgery (BASE), after the ketamine bolus (T1) and the MgSO_4_ bolus (T2), during the skin incision (SKIN), the osteotomy (OSTEOTOMY) and skin suturing (SUTURE). In the postoperative period, the short form of Glasgow Composite Pain scale (SF-CMPS) was used to assess pain at 30, 60, 120, and 180 min after extubation (Post30, Post60, Post120, and Post180, respectively). The main blood electrolytes (Mg^2+^, Ca^2+^, Na^+^, K^+^) were analyzed at BASE, T2, OSTEOTOMY, SUTURE and T3 (one hour after stopping MgSO_4_ infusion). Number of rescue analgesia and administration times were recorded both in the intra-and postoperative period.

**Results:**

In K group 7 out of 10 dogs required intraoperatory rescue analgesia compared to MK group (3/10). Furthermore, mean arterial pressure (MAP) and heart rate (HR) were significantly higher at OSTEOTOMY compared to BASE time in both groups. In the postoperative period, at T120, ICMPS-SF score was higher in K group than MK group.

**Conclusion:**

The administration of MgSO_4_ could guarantee better analgesia in the perioperative period in dogs undergoing TPLO, performing a synergistic action with ketamine.

## 1 Introduction

The magnesium is an important cation that involved in several biological process such as gating of calcium channels, hormone receptor binding, transmembrane ion flux, but also muscle contraction, neuronal activity, control of vasomotor tone, cardiac excitability and neurotransmitter release (1). In all these functions, magnesium acts like a physiologic calcium antagonist (2). In recent years, it has been hypothesized that magnesium may have an analgesic action (3). However, it does not have a direct antinociceptive action, but it inhibits the entry of Ca^2+^ ions into cells, blocking NMDA (N-Methyl-D-Aspartate) receptors, with a consequent indirect analgesic effect (4). Specifically, NMDA receptors are ion channels expressed by the nervous system (NS), whose activation is directly involved in the induction of central sensitization and potentiation of short-and long-lasting pain (5, 6). NMDA receptors are opened by pro-algogenic neuropeptides such as glutamate and substance P, which induce membrane depolarization. In contrast, NMDA antagonists, like magnesium and ketamine, act non-competitively block the ion channel, preventing its opening (7, 8).

Despite these assumptions, over the last 25 years, the use of perioperative constant rate infusion (CRI) of magnesium sulphate (MgSO_4_) in human medicine has led to conflicting results. Several studies state that the administration of MgSO_4_ infusion guarantees better management of acute pain and reduces the dose of opioids required in the intra-and postoperative period both in soft tissue surgeries, such as laparoscopic cholecystectomies, gynecological procedures and in orthopedics (e.g., lower limb orthopedic surgeries) (9–11). On the other hand, Seong-Hoon et al., state that the use of MgSO_4_ has no analgesic action in patients undergoing hysterectomy (12). Similarly, Durmus et al., demonstrated that patients, undergoing elective surgery who received infused magnesium, required elevated sevoflurane minimum alveolar concentrations (MAC) (13).

In veterinary medicine, references about the use of MgSO_4_ are very few and incomplete (14). Many studies describe its application in association with local anesthetics, in order to obtain longer duration of regional analgesia (15–18). As regards its endovenous use, the studies performed are not encouraging. Roja et al., demonstrated that the administration of magnesium in dogs undergoing ovariohysterectomy does not reduce the MAC of isoflurane and does not improve postoperative pain management (19). In agreement with them, Johnson et al., studied the effectiveness of magnesium infusion in association with propofol, not highlighting any advantage in its use (20). Differently, Anagnostu et al., evaluated the thiopental dose-sparing effects of magnesium sulphate in dogs, demonstrating that, administered at a dosage of 12 mg/kg/h, it is effective to reduces the volume of thiopental used, but inducing secondary side effects (nausea and vomit) (21).

Recently, other authors hypothesized that, since ketamine and magnesium act on different sites of the NMDA receptor, the combination of both drugs could lead to a synergic effect, obtaining excellent results (reduced amount of morphine administered, better quality of awakening and high satisfaction score during the first post-operative night) (22, 23). However, in the veterinary field, there are no scientific studies to prove it.

Based on the evidence described in human and veterinary medicine, the authors’ opinion is thata more suitable application of magnesium sulphate could be in association with another NMDA antagonist, such as ketamine, to enhance its efficacy. For this purpose the objective of this study was to evaluate the trans-surgical and post-surgical analgesic efficacy of MgSO_4_ in combination with ketamine in dogs under TPLO. Main physiological parameters were monitored during the intraoperative period, and, at the end of the procedure, the short form of Glasgow Composite Pain scale was used to evaluate postoperative pain (24). Furthermore, to identify any electrolyte imbalances, the blood level of Mg^2+^ and main electrolytes were monitored by blood gas analysis at different times of the study. Our hypothesis is that the use of MgSO_4_ may play a synergistic role with ketamine in enhancing intra-and postoperative analgesia in dogs affected by acute somatic pain.

## 2 Materials and methods

This randomized, prospective, blinded clinical study was approved by the Ethics Committee for Clinical Studies on Animal Patients of the University of Camerino, Italy (Prot. 06/2022). Furthermore, all owners were informed about the study and signed an appropriate consent form.

### 2.1 Animals

This study involved twenty adult mixed breed dogs (Table 1), conducted at the University Veterinary Teaching Hospital of Camerino between September 2022 and June 2023, to undergo TPLO (Tibial Plateau Leveling Osteotomy) surgery for ligament rupture cranial cruciate. All animals underwent physical examination and blood tests, including complete blood count, biochemistry, and coagulation profile. Exclusion criteria were: aggressive subjects, presence of pain not related to the above-mentioned orthopedic disease, dogs with coagulopathies and/or cardiovascular, hepatic, and renal diseases, pregnant females and obese and/or cachectic dogs (BCS < 3/5 or BCS > 3.5/5). Subjects with cranial cruciate ligament rupture requiring surgical resolution, free of other pathologies, assessed as ASA (American Society of Anesthesiologists) classification class II (25).

**Table 1:** Distribution of dog breeds in the two study groups undergoing tibial plateau leveling osteotomy.

Breed	K group	MK group
Mixed breed	1	3
American bull	1	1
Rottweiler	0	1
Golden retriever	2	2
Labrador retriever	1	1
Siberian husky	2	0
Gordon setter	0	1
Staffordshire bull terrier	0	1
Boxer	1	0
Shiba Inu	1	0
English pointer	1	0

K, group that received ketamine; MK, group that received ketamine plus MgSO_4_.

### 2.2 Anesthetic protocol

All animals were fasted for 12 h before surgery, while free access to water was maintained.

All dogs were premedicated with methadone (0.3 mg/kg; Semfortan®, Dechra Italia; 10 mg/mL) administered intramuscularly (IM). Then, the cephalic vein was cannulated for intravenous (IV) administration of medications and fluids (Ringer Lactate solution, 5 mL/kg/h; B Braun, Italy). Thirty minutes before the start of surgery and every 90 min until the end of surgery, cefazolin sodium (20 mg/kg, Cefazolin, Zoetis S.r.l.) was injected intravenously (IV).

During the induction of general anesthesia, patients were preoxygenated with pure oxygen by face mask, then, IV propofol (3–5 mg/kg; Proposure®, Boehringer Ingelheim Animal Healt Italia S.p.A; 10 mg/mL) was administered until adequate muscle relaxation was achieved (muscle relaxation of the limbs, relaxation of the jaws, and loss of the pedal reflex). All dogs were intubated, connected to a circular breathing system and maintained under general anesthesia with isoflurane (IsoFlo, Zoetis S.r.l, Milan, Italy) in a mixture of oxygen and air, maintaining an inspired fraction of O_2_ between 65 and 70% (FiO_2_ 65–70%). These were also mechanically ventilated in volume-controlled mode (Datex-Ohmeda S/5 Avance, Madison, Wisconsin, USA). The settings were 12 mL/kg tidal volume (VT), inspired to exhaled ratio (I:E ratio) 1:2, respiratory rate (RR) variable on the basis of end-tidal carbon dioxide (EtCO_2_ = 35–45 mmHg) and Positive End Expiratory Pressure (PEEP) = 0 cmH_2_O.

When an adequate anesthetic depth level was achieved, a 22-gauge cannula was inserted into the dorsal pedal artery for measurement of systolic, mean, and diastolic blood pressure (SAP, MAP, and DAP, respectively; mmHg) and collection of arterial blood samples for the evaluation of blood electrolytes. The VetStat Electrolyte Blood Gas Analyzer was used for the analysis of Na^+^, K^+^, Ca^2+^ and Cl^−^ (Idexx VetStat 8 Plus Cassettes, Idexx Laboratories Italia Srl, Italy; Idexx VetStat Ionized Calcium Cassettes, Idexx Laboratories Italia Srl, Italy) and the Catalyst One Analyzer (single Mg slice, Idexx Laboratories Italia Srl, Italy). The pressure transducer was positioned at the level of the right atrium and zeroed to atmospheric pressure. A multiparametric monitor (BeneView T8, Mindray Medical S.r.l) was used to assess the main cardiovascular and respiratory parameters. Specifically, the following data were manually collected every five minutes during the entire procedure: heart rate (HR; beats/min); invasive SAP, MAP and DAP, peripheral capillary oxygen hemoglobin saturation (SpO_2_; %); RR (breath/min), peak and plateau inspiratory pressure (Ppeak and Pplat, cmH_2_O), EtCO_2_, end-tidal concentration of isoflurane (EtIso; %); inspired fraction of isoflurane (FiIso; %); minimum alveolar concentration of isoflurane (MAC; %); and temperature (T, °C). A possible lightening of the anesthetic plan was clinically evaluated (presence of the eyelid reflex and position of the eyeball) and was managed by administering 1 mg/kg of propofol IV or increasing the FiIso. In case of hypotensive events continuing for 1 min (MAP <60 mmHg), a bolus of crystalloids was first administered (5 mL/kg in 5 min) (26). If subjects were not responsive to fluids, a CRI of norepinephrine was started (0.1–0.2 μg/kg/min) (Norepinephrine Tartrate 2 mg/mL, S.A.L.F., Bergamo, Italy). Moreover, if HR was <50 beats/min for more than one minute, atropine sulphate (0.01 mg/kg; Atropine Sulfate 1 mg/mL, Fatro Spa, Italy) was administered IV.

At the end of the procedure, dogs were awakened and monitored in their cage and the affected limb was bandaged with a modified Robert-Jones bandage. After extubation, hypothermia (T < 37°C) and severe/moderate hypoxemia (SpO_2_ < 95%) were managed with suitable thermal support and supplementary oxygen (flow by, face mask or CPAP helmet), respectively. In addition, one hour after the extubation, all dogs received a subcutaneous administration of non-steroidal anti-inflammatory (carprofen 4 mg/kg; Rimadyl 50 mg/mL, Zoetis S.r.l) The TPLO performed in this study were performed by the same expert surgeon.

### 2.3 Study protocol

After the pre-anesthesiologic examination, all dogs were sorted into two groups using a random number generator (Microsoft® Excel®; Microsoft 365 MSO 2021, Italy):

- K group (10 dogs) received a bolus of ketamine (0.5 mg/kg), administered IV over 2 min, followed by a CRI of the same drug (1 mg/kg/h).
- MK group (10 dogs) received a bolus of ketamine administered at the same dosage described in the previous group. In addition, at the end of the bolus and at the same time as starting the ketamine infusion, MgSO_4_ (50 mg/kg) was administered IV over 15 min. Subsequently, a CRI of MgSO_4_ was also applied (15 mg/kg/h). Both infusions were stopped at the end of surgery (19).

#### 2.3.1 Intraoperative assessment

The main hemodynamic and respiratory parameters (HR, RR, SAP, MAP, DAP, SpO_2_, MAC, T°, EtCO_2_) were recorded in both groups at BASELINE (10 min before the ketamine bolus), T1 (end of ketamine bolus), T2 (end of the MgSO_4_ bolus in the MK group and 15 min after the ketamine bolus in the K group), SKIN (skin incision), OSTEOTOMY (the corrective osteotomy), and SUTURE (suture of the skin plane). For both groups the end of the infusions coincided with the SUTURE time.

Furthermore, in the intraoperative period, arterial blood samples were obtained from the dorsal pedal artery for the evaluation of the main electrolytes (Mg^2+^, Ca^2+^, Na^+^, K^+^) by blood gas analysis. The samples were collected at BASELINE, T2 and SUTURE times. In MK group, a further sampling was performed at the OSTEOTOMY time, in order to monitor the blood Mg^2+^ concentration. During the surgery, an increase in HR or MAP greater than 20% above T2 for more than one minute was considered a nociceptive autonomic response to surgical stimulation; in this case, the CRI of ketamine was increased to 2 mg/kg/h. However, if the above-mentioned parameters did not fall within the expected ranges in the following 10 min or a second nociceptive peak was recorded, a bolus of fentanyl (1 μg/kg IV, Fentadon, Dechra Italia) was administered and the data was noted (25). If the fentanyl bolus was not sufficient, a CRI of this was initiated, and the patient was excluded from the study. The duration of surgery (from the first incision to the end of the skin suture) and anesthesia (duration of isoflurane administration) were recorded. Additionally, any anesthetic or surgical complications during the procedure and extubation time (from the end of anesthesia to removal of the endotracheal tube) were noted.

#### 2.3.2 Postoperative assessment

Physiological parameters (HR, RR, MAP and T°) were recorded 30, 60, 120 and 180 min after extubation (POST30, POST60, POST120 and POST180, respectively). Specifically, as regards MAP, it was monitored non-invasively (SunTech Vet 25 Blood Pressure Monitor, Ancyon Italia Srl, Italy) using specific cuffs based on the diameter of the dog’s limb according to the indications of the manufacturer (Suntech Bayonet Blood Pressure Cuffs, Alcyon Italia Srl, Italy) positioned in all subjects at the level of the radial artery (left forelimb). The measurements were taken keeping the limb raised, at the level of the cardiac area. Before manipulating dogs, the short form of the Glasgow Composite Measure Pain Scale (SF-CMPS) was used to assess the presence of pain in this study phase. As indicated by the pain scale itself, it was excluded the question B relating to walking ability, since all subjects had orthopedic pathology. The score attributable to pain was therefore not 6/24 but, 5/20. If dogs reached this score, a bolus of methadone (0.3 mg/kg) would be administered (IM) and the monitoring would be stopped. The time of administration of the rescue analgesia was also recorded. In addition, a final blood gas analysis was performed one hour after the end of the MgSO_4_ infusion (T3) in order to evaluate the blood concentration of Mg + and the adequacy of electrolyte balance.

## 3 Statistical analysis

MedCalc 9.0 software (MedCalc version 9.2.10) was used to perform the statistical analysis. All data resulted normally distributed based on the Kolmogorov–Smirnov test and they were compared between groups and at different study times. Cardinal data were analyzed with an independent t-test to compare between groups. ANOVA for repeated measures was used to compare the study times within group. Ordinal variables were analyzed with Kruskal-Wallis test to obtain a comparison between the two groups and Friedman test was used to perform a comparison between the study times within each group. Results for cardinal variables are presented as mean ± standard deviation and ordinal variables as median (minimum – maximum). Instead, for the yes/no variables (intra-and postoperative rescue analgesia), Fisher’s exact test was used. A *p* value <0.05 were considered statistically significant.

## 4 Results

**Figure 1:**
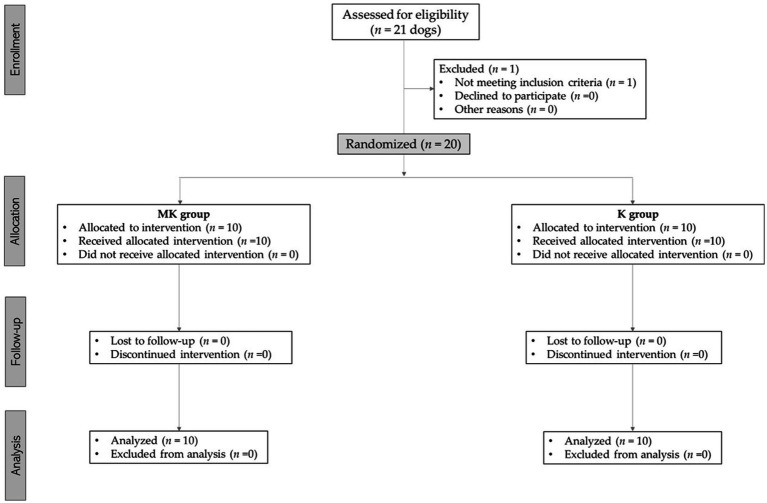
Consolidated standards of reporting trials (CONSORT) flow diagram for subjects included in this study.

Twenty-one dogs were considered for this study. During the pre-anesthesiologic evaluation, one subject was excluded for not meeting the inclusion criteria (cardiopathy not previously investigated), as reported according to the CONSORT Statement 2010 for randomized clinical trials (27) (Figure 1).

There were no significant differences in the age (MK = 6.6 ± 2.67 years; K = 5.3 ± 2.66 years) and weight (MK = 28.1 ± 9.03 kg; K = 27.67 ± 9.34 kg) of dogs included in the study. Likewise, no differences were found in the duration of surgery (MK = 94.1 ± 8.6 min; K = 88 ± 10.6 min) and anesthesia (MK = 156.2 ± 36.3 min; K = 140.9 ± 36.8 min).

### 4.1 Intraoperative assessment

There were no significant differences in RR, EtCO_2_, MAC and T between the two groups at all times of the study. The HR, in K group, was significantly lower at BASE (*p* = 0.043), T1 (*p* = 0.042), T2 (*p* = 0.048) and SKIN (*p* = 0.036) compared to OSTEOTOMY time. In the same way, in both groups, the MAP was statistically higher at OSTEOTOMY compared to BASE, T1, T2 and SKIN times (*p* < 0.01). Moreover, at SUTURE, it was higher than BASE [K (*p* = 0.034); MK (*p* = 0.048)] and T1 [K (*p* = 0.044); MK (*p* = 0.035)] times. In both groups, DAP was higher at OSTEOTOMY and SUTURE compared BASE, T1, T2 and SKIN times (*p* < 0.01) (Table 2).

**Table 2:** Mean ± SD of cardiovascular and respiratory parameters at different time points during the study in dogs undergoing tibial plateau leveling osteotomy (TPLO) with magnesium sulphate and ketamine.

Parameter	Group	Base	T1	T2	Skin	Osteotomy	Suture
HR (beats/min)	K	68.9 ± 16.6°	68.2 ± 10.9°	69.7 ± 9.8°	67.4 ± 9.3°	92 ± 25.6	78.4 ± 19.7
	MK	78.3 ± 23.3	73.2 ± 23	66.6 ± 21.4	66 ± 17.2	83.7 ± 24.8	72.3 ± 27.9
RR (breaths/min)	K	10.1 ± 3.5	9.6 ± 4.5	10.4 ± 4.7	10.4 ± 4.7	12.7 ± 5.6	13.5 ± 5.5
	MK	13.1 ± 4.1	11.4 ± 4.08	11.7 ± 4.4	11.7 ± 4.4	12.8 ± 3.6	13 ± 3.7
SAP (mmHg)	K	114.2 ± 28.5	117.7 ± 30.6	114.2 ± 31.5	122.3 ± 34	135.8 ± 26.4	126 ± 26.7
	MK	107 ± 19.07	101.9 ± 16.7	117.8 ± 11.6	109.4 ± 21.5	135.4 ± 27.8	125.8 ± 12.4
(mmHg)	K	73.8 ± 19.3°^*^	74.6 ± 24.1°^*^	78.1 ± 25.2°	78.3 ± 22.2°	100.3 ± 21.2	90 ± 23.1
	MK	76.7 ± 17.9°^*^	72.8 ± 14.2°^*^	84.6 ± 15.08°	83.3 ± 18.5°	115.6 ± 26.5	96.5 ± 11.8
DAP (mmHg)	K	57.5 ± 19.4°*	53.7 ± 18.1°*	57.6 ± 19.4°*	60.2 ± 18.1°*	78.7 ± 17.3	72.7 ± 22.7
	MK	54 ± 7.7°*	50 ± 10°*	61.4 ± 7.7°*	57.2 ± 13.4°*	85.2 ± 28.9	79.2 ± 11.9
ETCO_2_ (mmHg)	K	36.4 ± 7.1	34.8 ± 3.08	35.9 ± 3.1	37.6 ± 2.01	40.7 ± 3.09	40.1 ± 5.7
	MK	40.5 ± 7.1	38.9 ± 5.7	38.2 ± 4.8	38.7 ± 4.5	37.3 ± 5.9	40.3 ± 4.5
MAC (%)	K	0.97 ± 0.2	0.95 ± 0.1	0.97 ± 0.1	0.94 ± 0.2	0.98 ± 0.3	0.90 ± 0.2
	MK	0.75 ± 0.1	0.78 ± 0.1	0.85 ± 0.2	0.86 ± 0.2	0.92 ± 0.2	0.89 ± 0.2
T (°C)	K	37.08 ± 0.6	36.8 ± 0.8	36.7 ± 0.7	36.5 ± 0.9	36.3 ± 1.1	36.2 ± 1.06
	MK	37.1 ± 0.6	37.05 ± 0.6	36.5 ± 0.7	36.4 ± 0.7	36.04 ± 0.8	35.4 ± 0.8

Significant differences are indicated by p° compared to OSTEOTOMY and p* compared to SUTURE times. K, group that received ketamine; MK, group that received ketamine plus MgSO_4_; BASELINE, 10 min before the ketamine bolus; T1, end of ketamine bolus; T2, end of the MgSO_4_ bolus in the MK group and 15 min after the ketamine bolus in the K group; SKIN, skin incision; OSTEOTOMY, the corrective osteotomy; SUTURE, suture of the skin plane.

**Figure 2:**
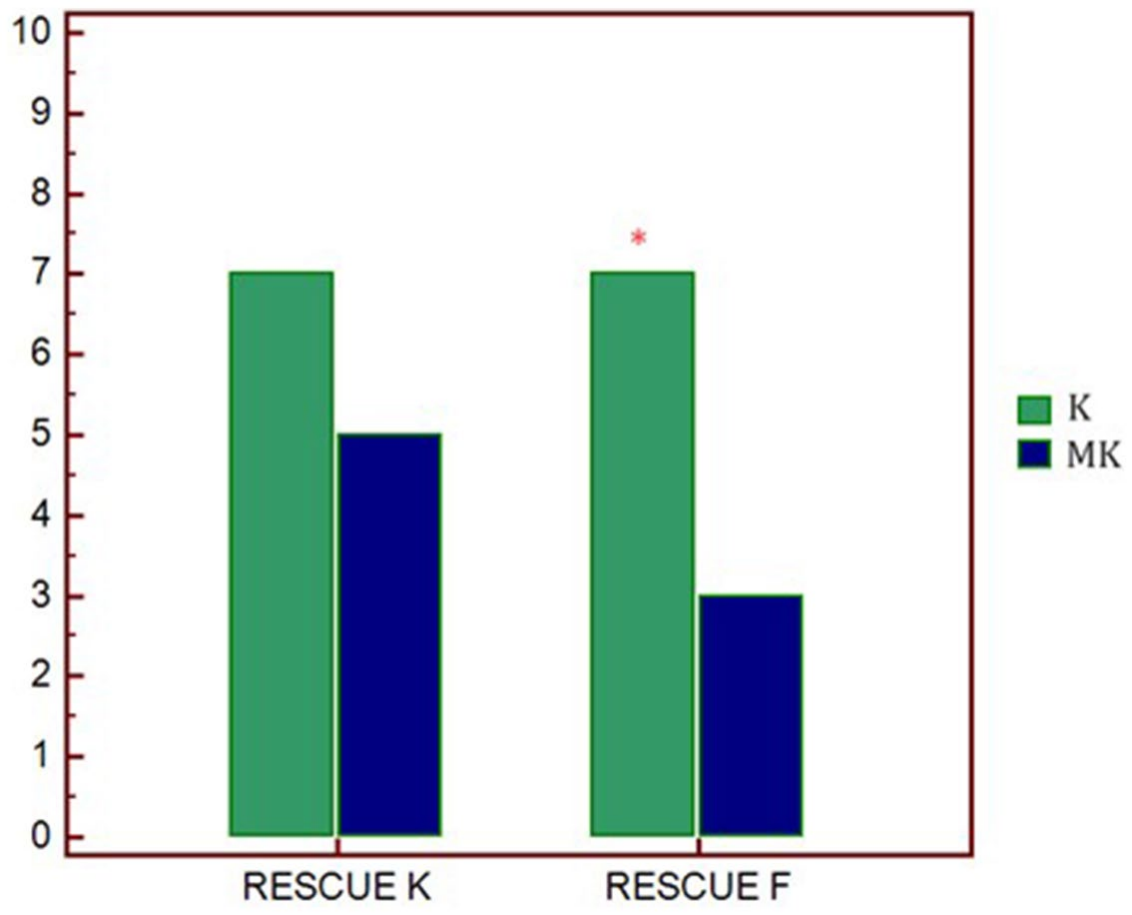
Graphical representation of the request for rescue analgesia in the two study groups. p* < 0.01 differences between groups. K, group that received ketamine; MK, group that received ketamine plus MgSO_4_; RESCUE K, number of ketamine rescues administered; RESCUE F, number of fentanyl rescues administered.

Regarding the rescue analgesia administered, in both groups, it was necessary to increase the CRI of ketamine (7 out of 10 dogs). However, in K group, 7 out of 10 patients also required a bolus of fentanyl while, in the MK group, only 3 out of 10 (Table 3; Figure 2).

**Table 3:** Number of dogs requiring ketamine and fentanyl rescue analgesia during the intraoperative period.

Intraoperative rescue	Ketamine	Fentanyl
K (n°)	7/10	7/10
MK (n°)	5/10	3/10^*^

p*p* < 0.01 differences between groups. K, group that received ketamine; MK, group that received ketamine plus MgSO4.

### 4.2 Postoperative assessment

**Figure 3:**
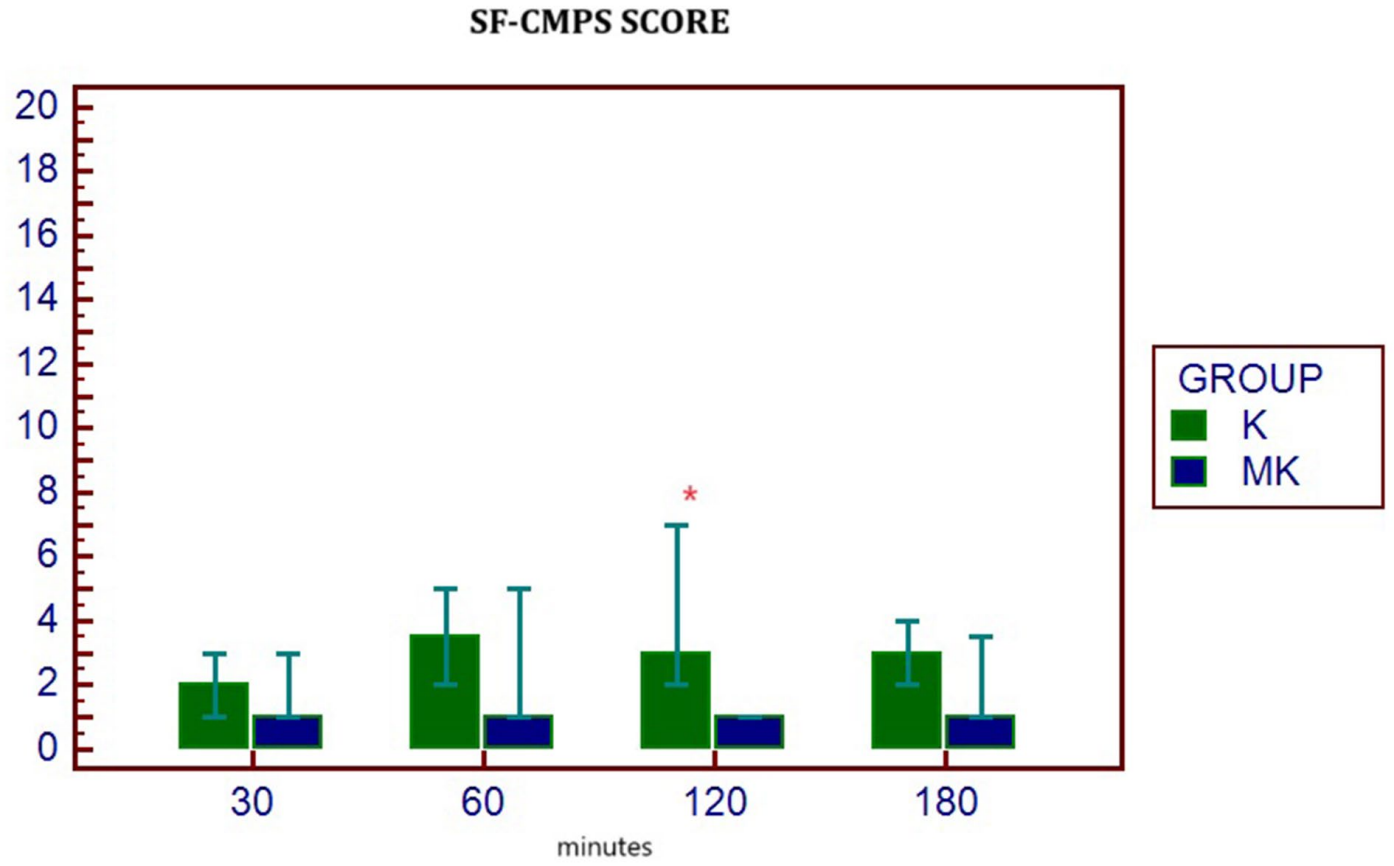
Median (min – max) of SF-GMPS values in the two groups at different postoperative study times. p* 0.047 differences between groups. K, group that received ketamine; MK, group that received ketamine plus MgSO_4_; POST30, POST60, POST120, and POST180 = 30, 60, 120, and 180 min after extubation, respectively.

The mean ± SD of physiologic parameters are reported in Table 4. HR, MAP and RR did not show significant differences between groups at any study times. At T180 time, the number of patients who required rescue analgesia was significantly greater in the K group (8/10) than MK group (4/10) (Table 4). In agreement with this result, 120 min after extubation, the SF-CMPS score was significantly higher in the K group compared to MK (Figure 3).

**Table 4:** Main postoperative parameters monitored during tibial plateau leveling osteotomy (TPLO) in the Ketamine and MgSO_4_ plus Ketamine groups.

Parameters	Groups	T30	T60	T120	T180
HR (beats/min)	K	89.3 ± 16.1	95.8 ± 14.4	94.8 ± 17.6	96.5 ± 12.02
	MK	96.5 ± 14.9	101.6 ± 17.4	94.5 ± 16.2	104.8 ± 16.03
(mmHg)	K	106.5 ± 20.1	110.4 ± 17.1	109.5 ± 26	84 ± 8.4
	MK	107.1 ± 11.5	111.6 ± 14.1	119.5 ± 26.03	103.4 ± 20
RR (breaths/min)	K	26.4 ± 11	31.5 ± 5.9	33.6 ± 8.2	30 ± 8.4
	MK	28.5 ± 13.9	32.5 ± 13.9	38.6 ± 13.5	40 ± 14.1
RESCUE (n°)	K	0/10	3/10	4/10	8/10
	MK	0/10	2/10	3/10	4/10^*^

Significant differences (p* 0.047) between groups. K, group that received ketamine; MK = group that received ketamine plus MgSO_4_.

### 4.3 Blood electrolyte assessment

**Figure 4:**
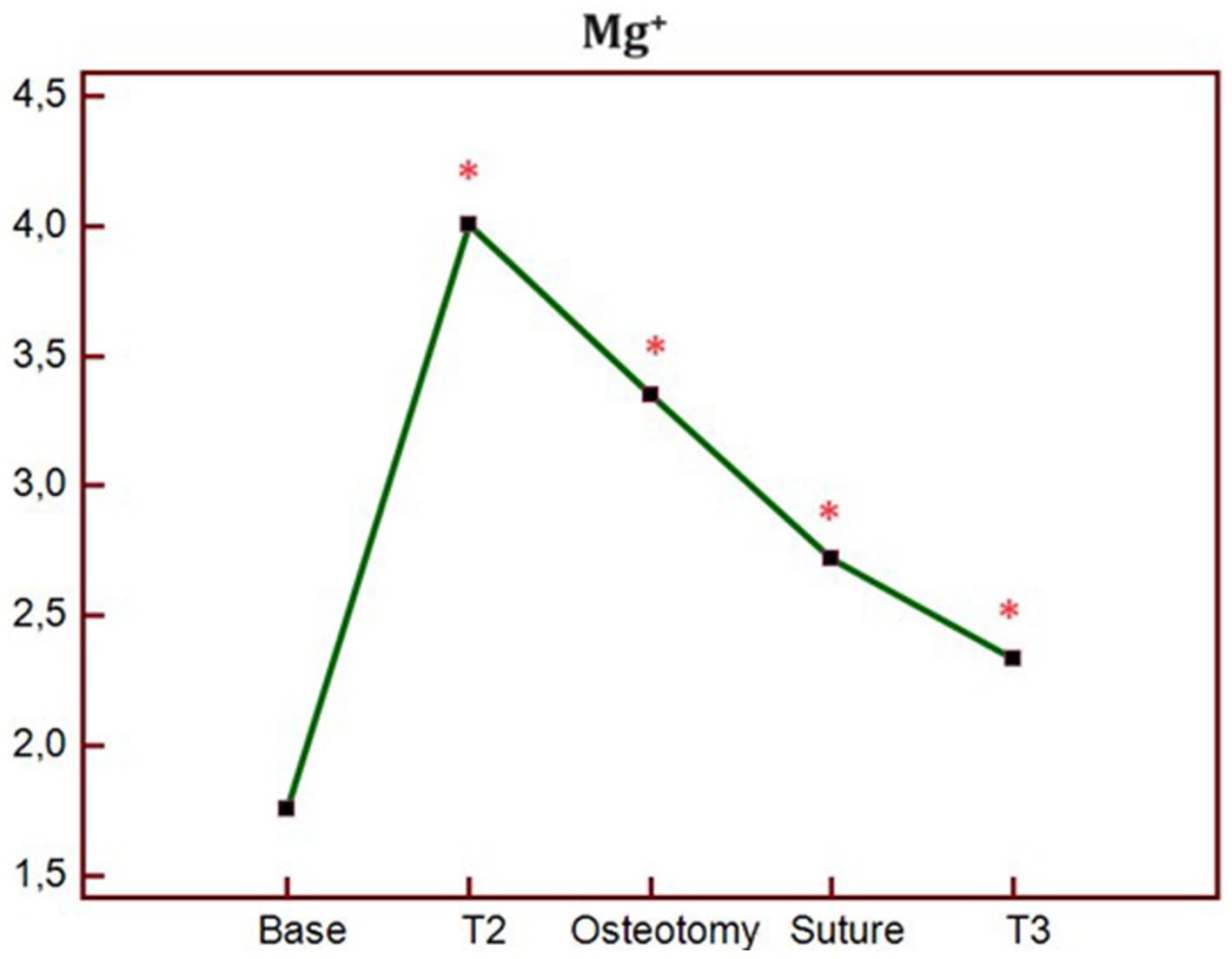
Trend of blood concentration of Mg + at different times of study in MK group. p* < 0.05 differences compared to BASE. BASELINE, 10 min before the ketamine bolus; T1, end of ketamine bolus; T2, end of the MgSO_4_ bolus in the MK group and 15 min after the ketamine bolus in the K group; SKIN, skin incision; OSTEOTOMY, the corrective osteotomy; SUTURE, suture of the skin plane.

The analysis of blood electrolytes showed no statistically significant differences in the K group at all study times. In contrast, in the MK group, Mg^2+^ was significantly higher at T1, T2, OSTEOTOMY, SUTURE, and T3 times compared to BASE (Figure 4).

## 5 Discussion

This study aimed to evaluate the synergistic efficacy of magnesium sulphate, in combination with ketamine, in dogs undergoing orthopedic surgery. Our results showed that there were fewer analgesic rescues in MK compared to the K group. Furthermore, in the postoperative period, patients who received MgSO_4_ plus ketamine obtained a lower value on the SF-CMPS. To the authors’ knowledge, this is the first study to confirm the potentiating action of magnesium on the antinociceptive efficacy of ketamine in dogs.

A previous study performed in dogs undergoing ovariohysterectomy demonstrated that MgSO_4_ has no benefit in intraoperative pain management (19). Our results are partially in agreement with Rioja et al. (19). In fact, we highlighted that the requirement for rescue analgesia was lower in the group that received both MgSO_4_ and ketamine, however, we still recorded a significant increase in MAP, DAP and HR during the osteotomy. Our hypothesis is that, in our study, unlike Rioja et al., we did not use MgSO_4_ alone, but in association with another NMDA antagonist. The enhanced synergism between the two provided better analgesic coverage and, therefore, a reduced need for rescue interventions. This agrees with Queiroz-Castro et al. who demonstrated that the magnesium-ketamine association provides a better sparing effect on isoflurane compared to magnesium alone in goats subjected to experimental nociceptive stimulation (28). The actual mechanisms of interaction between the two drugs are not fully understood, however, experimental model studies have shown that the synergy between MgSO_4_ and ketamine is mainly due to the different mechanisms of action of the two molecules (29, 30). In fact, magnesium blocks the flow of calcium and antagonizes the NMDA receptor channels, while ketamine binds to the phencyclidine binding site of NMDA receptors and modifies them through allosteric mechanisms. Furthermore, it is known that ketamine interacts with calcium and sodium channels, dopamine receptors, cholinergic transmission, noradrenergic and serotonergic reuptake, carrying out opioid-related and anti-inflammatory effects (31). On the other hand, magnesium has been shown to reduce the activity of other presynaptic and postsynaptic calcium channels, modulate the release of some neurotransmitters and influence sodium and potassium currents, interfering in the physiological action of membrane potentials (1, 32). These data support our hypothesis, however, probably, during severe pain stimulation (OSTEOTOMY), this association is not sufficient to provide a suitable antinociceptive action (19, 28).

Therefore, the results obtained show that magnesium, although carrying out a synergistic strengthening action with ketamine, is not sufficient to counteract acute nociceptive phenomena during orthopedic surgery. In our study we chose an increase in the CRI of ketamine to 2 mg/kg/h as the first rescue analgesia, however, it would be interesting to evaluate whether, by using higher doses of this drug from the beginning of the procedure, we could modulate and improve the action of magnesium. Higher concentrations of ketamine could have bound to the phencycline binding site more effectively, thus allowing an increase in Mg^2+^ concentration to improve the quality of receptor blockade. This would also support the hypothesis of Vujovic et al., who stated that magnesium has greater analgesic efficacy if ketamine is administered before it. On the other hand, it is weaker when magnesium is administered first. The explanation for this concept could be that magnesium is able to temporarily block NMDA channels, preventing the binding of ketamine and, therefore, its antinociceptive action (29, 30).

NMDA receptors play a key role in the modulation of pain and different inflammatory responses, preventing central sensitization caused by peripheral nociceptive stimulation (33). Having recognized the different mechanism of action of ketamine and magnesium at the level of NMDA receptors, their synergistic action still needs to be further investigated to better understand the mechanisms. From the results obtained in the intraoperative period we can therefore affirm that the magnesium/ketamine association provided good analgesia during the surgical procedure, such as to reduce the request for rescue analgesia, however, it seems not to be able to fully regulate the hemodynamic response to acute nociceptive stimuli and the consequent sympathetic activation.

Regarding the results obtained in the post-operative period, the subjects who had received an infusion of MgSO_4_ required rescue analgesia later than the K group, furthermore, they showed reduced SF-GMPSG scores at all times of the study and significantly lower at 120 min after extubation. In addition, at 180 min, 4/10 patients in the MK group received rescue analgesia, compared to the K group, in which the majority (8/10) required it. Our results agree with previous studies in which the authors demonstrated the effectiveness of NMDA antagonists on the management of postoperative pain and on the prevention of the development of pathological alterations of the nociceptive pathways, with consequent manifestation of hyperalgesia and allodynia. Kanta et al., in an experimental study on a mouse model, showed for the first time how the administration of magnesium inhibits the expression of the glutamate ionotropic receptor NMDA type subunit 1 (Grin1), thus reducing the development of hyperalgesia and chronic postoperative pain (34). Similarly, other authors, described the effectiveness of NMDA antagonists in the management of chronic pain, favoring the significant reduction in the use of opioids in the postoperative period (35–37).

.As regards the choice of the dose of magnesium sulphate administered, there are no guidelines in veterinary medicine. For this reason, we chose the dosages previously used by Rioja et al. in their study (19). In addition, blood Mg^2+^ concentrations were monitored to understand its trend at different study times. The main risk that can be incurred by administering high concentrations of MgSO_4_ in bolus is secondary hypermagnesemia. This may cause nausea, vomiting, hypocalcaemia and cardiac arrhythmias. In our study, no side effects were highlighted at the chosen doses, however, blood concentrations significantly increased at T2 time, and then gradually decreased until T3 (although remaining significantly higher than the BASE time). Hypermagnesemia is easily managed by patients with normal renal function thanks to the rapid renal excretion of magnesium, however, it is important to know the hematobiochemical condition before choosing to administer this drug (21).

The authors considered some limitations in this study. The first is the absence of a group treated only with MgSO_4_. This would allow us to better understand the synergistic action carried out with ketamine rather than identifying the analgesic action of MgSO_4_ alone. Furthermore, it would have been interesting to monitor blood magnesium concentrations until BASE concentrations returned, however, it was not possible to perform further blood sampling due to the clinical nature of the study. Another limitation to consider is that the quality of the dogs’ awakening was not assessed. Considering the side effects that ketamine can induce (dysphoria, spasms, delirium) in the awakening phase, this component would be important to define, especially if we choose to use moderately high continuous infusion dosages (e.g., 2 mg/kg/h). Finally, we consider the absence of SAP and DAP evaluation in the postoperative period as a limitation of the study, since, for completeness, it would have been more appropriate to record all data relating to blood pressure.

In conclusion, the preliminary data presented in this study demonstrates for the first time the existence of a synergism between MgSO_4_ and ketamine in the management of pain in dogs undergoing TPLO surgery. In fact, the intraoperative co-administration of ketamine and MgSO_4_ seems to be more effective in reducing pain and opioid consumption than an analgesic protocol with ketamine alone. However, it is the authors’ opinion that, as already demonstrated, locoregional anesthesia represents the gold standard for pain management during this type of surgical procedure (38, 39). Furthermore, although a synergism between MgSO_4_ and ketamine was detected, this does not seem to be sufficient to completely manage severe intraoperative nociceptive stimuli, therefore, we believe that analgesic integration with MgSO_4_ infusion could represent a useful analgesic support mainly in the postoperative period and in the prevention of central sensitization following nociceptive stimulation. Increasing the number of cases and including a third group that will only be administered MgSO_4_ will help us get more data and better understand this topic.

## Data Availability

The raw data supporting the conclusions of this article will be made available by the authors, without undue reservation.
